# EasyCen: A Lightweight Framework for Centromere Localisation and Repeat‐Organisation Profiling in Telomere‐to‐Telomere Genomes

**DOI:** 10.1111/1755-0998.70176

**Published:** 2026-07-02

**Authors:** Yunyun Lv, Yanping Li, Jia Li, Xidong Mu

**Affiliations:** ^1^ Key Laboratory of Sichuan Province for Fishes Conservation and Utilization in the Upper Reaches of the Yangtze River, College of Life Science Neijiang Normal University Neijiang China; ^2^ Ministry of Agriculture and Rural Affairs, Guangdong Modern Recreational Fisheries Engineering Technology Center Pearl River Fisheries Research Institute, Chinese Academy of Fishery Sciences Guangzhou China

**Keywords:** centromere identification, comparative genomics, genome assembly, repeat architecture

## Abstract

Accurate identification of centromeres in telomere‐to‐telomere (T2T) genomes remains difficult due to the rapid evolution of centromeric repeats and their lack of conserved sequence features. In this study, we present EasyCen, a lightweight sequence‐based framework for centromere identification and repeat‐architecture profiling across various eukaryotes. Rather than relying on repeat annotation or homology, EasyCen recognises centromeres based on recurrent positional features of repetitive DNA. Besides centromere localisation, EasyCen incorporates a repeat‐pair profiling module for exploratory characterisation of internal repeat organisation. Benchmarking on *Arabidopsis thaliana* and *Mus musculus* showed high accuracy (typically > 85% coordinate overlap with published annotations) and substantially reduced runtime. Cross‐species analysis revealed a ‘beads‐on‐a‐string’‐like repeat pattern in both mouse and human centromeres, associated with GC‐ and CpG‐rich subdomains. EasyCen performs effectively without pre‐existing repeat libraries, making it particularly useful for large, repeat‐rich, or non‐model genomes. Our analyses further suggest that certain organisational features of centromeric repeats may recur across diverse eukaryotic lineages despite rapid sequence turnover.

## 1 Introduction

Long‐read sequencing technologies, including PacBio SMRT and Oxford Nanopore ultra‐long reads, have enabled telomere‐to‐telomere (T2T) assemblies across a rapidly expanding diversity of eukaryotes (Nurk et al. 2022; Rautiainen et al. 2023; Cheng et al. 2021; Li and Durbin 2024). Initially achieved in a few model organisms, such as human ( *Homo sapiens* ) (Nurk et al. 2022), mouse ( *Mus musculus* ) (Chaudhry et al. 2025), and thale cress ( *Arabidopsis thaliana* ) (Naish et al. 2021), these assemblies are now increasingly generated in non‐model species, including sandalwood ( *Santalum album* ) (Peng et al. 2024), rice ( *Oryza sativa* ) (Xie et al. 2025) and fish (Xue et al. 2021; Cui et al. 2025), through integrated long‐read, high‐throughput chromosome conformation capture (Hi‐C), and linked‐read strategies. This progress opens unprecedented opportunities to study chromosome organisation, repeat evolution, and genome architecture at high resolution.

Despite these advances, accurate identification and structural characterisation of centromeres remain challenging (Li and Durbin 2024). Centromeres are primarily composed of fast‐evolving tandem repeats and higher‐order repeat (HOR) arrays whose sequence composition, periodicity, and chromosomal distribution vary widely among species and even among chromosomes of the same genome (Naish et al. 2021; Melters et al. 2013; Wang et al. 2025). This apparent paradox—functional conservation despite rapid sequence divergence—suggests that centromere identity may be shaped more by large‐scale repeat organisation than by conserved sequence motifs (Nurk et al. 2022; Altemose et al. 2022; Logsdon et al. 2024). Existing methods heavily rely on repeat annotation or auxiliary epigenomic data, limiting their use across newly assembled genomes (Xu et al. 2024; Elphinstone et al. 2025). These tools prioritise either centromere localisation or repeat annotation, whereas integrated analysis of repeat organisation remains relatively limited.

Current computational tools have major limitations. Annotation‐based pipelines such as CentIER (Xu et al. 2024) and QuarTeT (Lin et al. 2023) require repeat libraries or extensive re‐annotation, resulting in high computational costs for large genomes. These workflows also rely on external tools for detection of tandem repeats and transposable elements, increasing complexity and bias. Sequence‐driven approaches like RepeatOBserver (Elphinstone et al. 2025) transform DNA into numerical representations and use Fourier analysis to detect periodicity, while tools such as TRASH (Wlodzimierz et al. 2023) and HiCAT (Gao et al. 2023) focus on monomer or HOR annotation rather than boundary definition. CDR‐Finder requires methylation data (Mastrorosa et al. 2024), limiting its applicability. Overall, existing methods differ substantially in their analytical focus, annotation requirements, and scalability, while integrated analysis of centromere localisation and repeat organisation remains relatively limited.

Building on existing k‐mer‐based centromere analyses (Altemose et al. 2022; Logsdon et al. 2024), we investigate whether positional features of repetitive sequences could provide complementary information for centromere identification and structural characterisation. We designed three metrics—periodicity, breadth, and clustering—to capture centromeric hallmarks from k‐mer positional distributions. Periodicity measures the regularity of tandem‐array spacing, breadth quantifies the fraction of chromosomes harbouring a given k‐mer, and clustering evaluates positional concentration along a chromosome. Together, these metrics distinguish centromere‐enriched repeats from dispersed elements using sequence‐derived information only.

In this study, we present EasyCen, a k‐mer‐based framework that integrates these metrics for de novo centromere identification and architecture profiling in T2T genomes. The workflow combines population‐weighted density profiling and adaptive boundary refinement to delineate centromeric regions without repeat annotation or experimental data. A repeat‐pair combinatorial model transforms positional relationships into a co‐occurrence matrix, enabling profiling of internal repeat structures and compositional transitions. Across eight different T2T genomes— *A. thaliana* , *M. musculus* , *H. sapiens* , maize ( *Zea mays* ), rice ( *O. sativa* ), *S. album* , large yellow croaker ( *Larimichthys crocea* ), and green alga ( *Chlorella sorokiniana* )—EasyCen achieved high sensitivity, completing centromere analysis within hours even for large mammalian genomes. We also found previously uncharacterised centromeric subregions enriched for GC and CpG islands in both human and mouse, suggesting a potential association between local sequence composition and structural heterogeneity within mammalian centromeres.

These results establish EasyCen as an integrated framework for centromere boundary definition, architecture profiling, and comparative analysis across lineages. As T2T assemblies expand across the tree of life, this approach enables the investigation of centromere evolution, repeat dynamics, and chromosome organisation. Although centromeric DNA sequences evolve rapidly, some organisational features—tandem periodicity, local homogenisation, and regional confinement—appear recurrently conserved. Rather than replacing existing annotation‐based pipelines, EasyCen is intended as a lightweight complementary framework for rapid centromere localisation and exploratory repeat‐organisation analysis in newly assembled genomes.

## 2 Results and Discussion

### 2.1 Overview of the EasyCen Workflow

**FIGURE 1:**
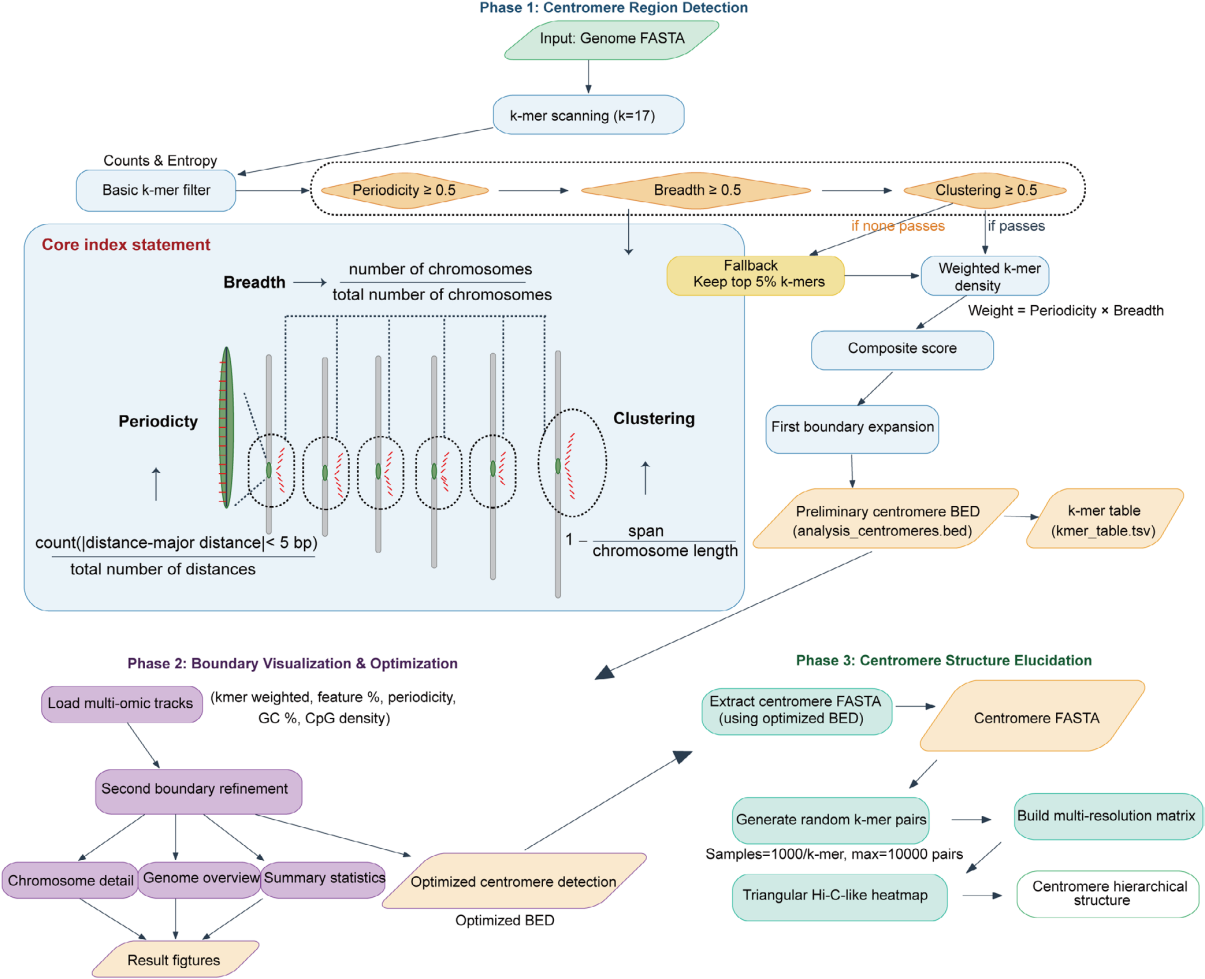
Conceptual framework and workflow of EasyCen for organisation‐guided centromere identification and repeat‐architecture profiling. EasyCen identifies centromeric regions directly from intrinsic positional features of repetitive sequences without requiring prior repeat annotation or auxiliary epigenomic data. In Phase 1, genomic sequences are decomposed into canonical k‐mers followed by abundance and entropy‐based filtering. Candidate centromeric k‐mers are evaluated using three organisation‐oriented metrics: periodicity (regularity of tandem repeat spacing), breadth (distribution across chromosomes), and clustering (local positional confinement). These features are integrated into weighted k‐mer density profiles to generate preliminary centromeric intervals. In Phase 2, boundary visualisation and refinement are performed using multiple sequence‐derived features, including weighted k‐mer density, periodicity, GC content, CpG density, and feature enrichment statistics, enabling optimisation of centromere coordinates and chromosome‐level summaries. In Phase 3, the internal centromeric organisation is reconstructed using repeat‐pair positional co‐occurrence analysis. Randomised k‐mer pairs extracted from optimised centromeric regions are transformed into Hi‐C‐like co‐occurrence matrices, allowing exploratory profiling of repeat layering, compositional transitions, and hierarchical centromeric structure. The central panel illustrates the conceptual basis of the three intrinsic organisational metrics used by EasyCen: periodic tandem spacing (periodicity), chromosome‐wide enrichment (breadth), and regional confinement of repetitive elements (clustering).

EasyCen is a fully sequence‐driven workflow consisting of three phases (Figure 1). It extracts centromeric signatures from k‐mer positional properties, avoiding dependence on repeat annotation or external data Table 1.

**TABLE 1:** Comparative evaluation of centromere localisation accuracy, computational efficiency, and repeat‐organisation profiling among EasyCen and existing frameworks in the thale cress ( *Arabidopsis thaliana* ) genome.

Chromosomes	CR (EasyCen)		CR (CentIER)		CR (RepeatOBserver)		CR (QuarTeT)	
1	14,660,000	17,240,000	14,841,146	17,128,917	14,730,000	17,120,000	14,294,878	17,560,046
2	3,750,000	6,060,000	3,800,000	6,045,215	4,055,000	5,770,000	3,785,663	6,280,846
3	13,300,000	16,730,000	13,590,000	15,990,000	13,830,000	15,845,000	13,597,093	15,812,055
4	3,190,000	7,210,000	4,200,000	7,040,000	4,765,000	6,365,000	4,203,833	7,062,957
5	10,840,000	14,760,000	11,200,000	14,400,000	11,885,000	14,265,000	11,772,414	14,461,783
Running time(s)	486		7,895		1,792		208	
Running time(m)	8		132		30		3	
Repeat‐organisation profiling	Yes		No		No		No	

In the ‘Analyse’ module, genomic sequences are converted into canonical k‐mers considering both strands. Low‐complexity and over‐dispersed sequences are removed by multi‐layer filtering. The retained k‐mers are evaluated using three metrics: periodicity (fraction of adjacent distances within 5 bp of the dominant repeat interval), breadth (proportion of chromosomes harbouring the k‐mer), and clustering (positional concentration). K‐mers meeting default thresholds (periodicity ≥ 0.5, breadth ≥ 50%, clustering ≥ 0.5) are designated as centromere‐like. These metrics effectively distinguish centromeric repeats from dispersed elements.

The workflow then calculates the centromere‐like k‐mer ratio within each window, improving discrimination from other repeat‐dense regions. Three additional features, namely GC content, CpG density, and unique k‐mer proportion, are profiled to build an integrated compositional landscape. Enriched windows are merged into preliminary centromeric blocks and classified as terminal, metacentric, or submetacentric based on a composite scoring framework.

Automated boundary refinement uses a Gaussian approximation of local feature distributions. In most centromeric regions examined, distributions approximated unimodal Gaussian‐like patterns; deviations in structurally heterogeneous regions were noted, but the model still provided objective boundary criteria. Users may also inspect distributions and manually adjust boundaries for complex genomes.

EasyCen additionally includes a k‐mer‐pair analysis module designed to summarise positional relationships among enriched repeat k‐mers. Pairwise genomic positions of repeat k‐mers within the identified centromeric regions are computed and converted into a positional co‐occurrence matrix akin to Hi‐C maps. This matrix provides an exploratory representation of repeat layering, homogenisation, domain partitioning, and compositional shifts within centromeric regions.

Similar to other k‐mer‐based approaches, EasyCen uses repetitive‐sequence enrichment as an initial signal for centromere detection. However, the framework additionally incorporates three positional metrics, namely periodicity, breadth, and clustering, to summarise repeat organisation patterns and support downstream structural analysis. Thus, the framework infers centromeric organisation directly from emergent repeat architecture rather than from repeat enrichment alone.

The computational efficiency of EasyCen stems from direct k‐mer indexing, avoiding whole‐repeat annotation and graph construction. Limitations include reduced sensitivity in genomes with weak tandem repeat enrichment, such as holocentric chromosomes, or where centromeres are characterised by low‐copy repeats or epigenetically specified states. Integration of chromatin data could improve delineation in such cases.

### 2.2 Benchmarking EasyCen Against Existing Centromere‐Identification Frameworks

**FIGURE 2:**
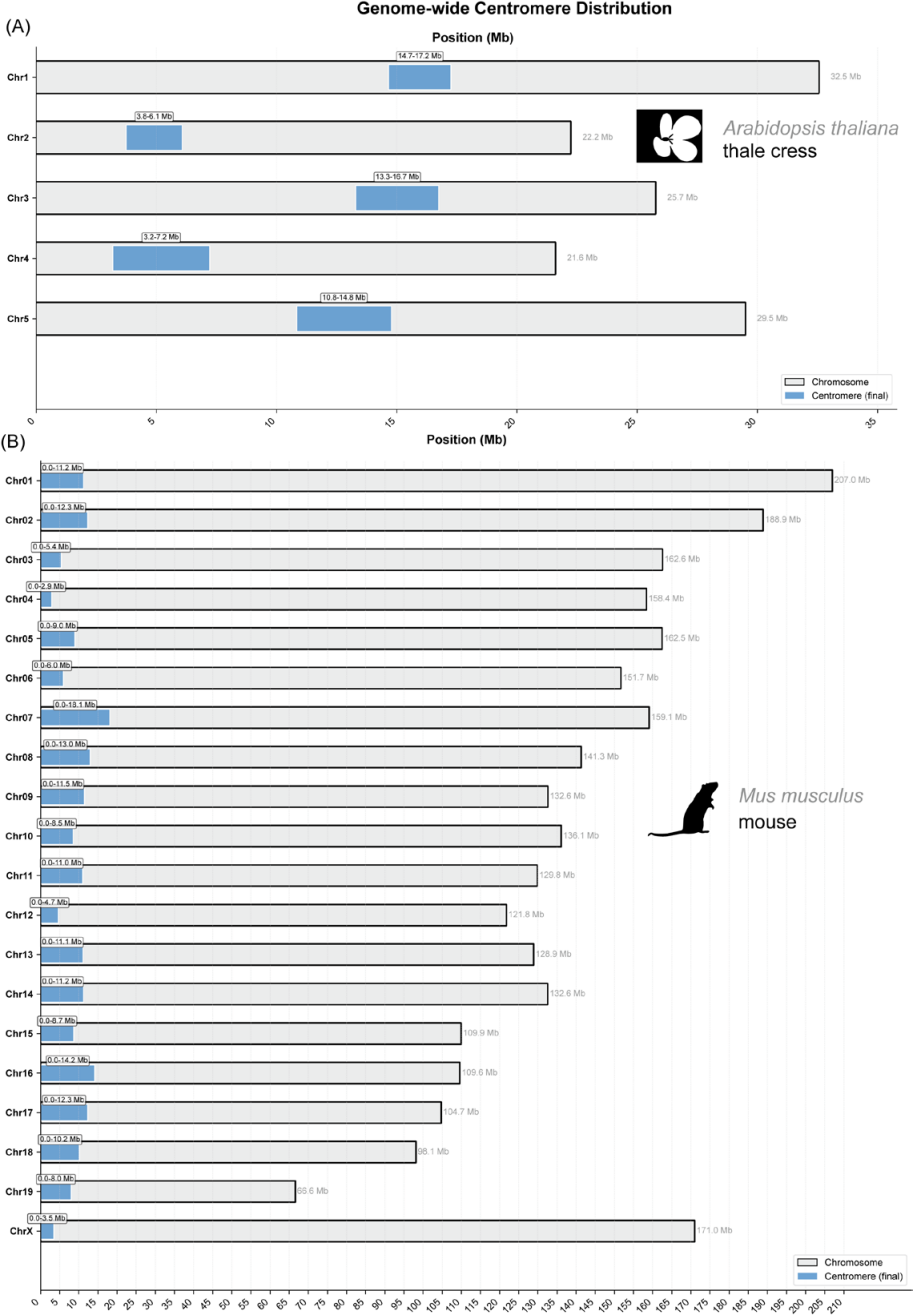
Representative centromere identification in plant and mammalian T2T genomes using EasyCen. Genome‐wide centromere distributions predicted by EasyCen in representative plant and mammalian telomere‐to‐telomere (T2T) assemblies. Grey horizontal bars indicate chromosome lengths, while blue regions represent optimised centromeric intervals identified by EasyCen. Coordinates of predicted centromeres are labelled above each region. (A) *Arabidopsis thaliana* (thale cress), showing characteristic regional centromeres distributed across all five chromosomes. (B) *Mus musculus* (mouse), illustrating predominantly pericentromeric repeat‐enriched regions near chromosome termini in the mouse T2T assembly.

We evaluated performance on *A. thaliana* (~132 Mb) and *M. musculus* (~2.77 Gb) to cover different genome sizes and repeat complexities. Predicted centromeric regions are summarised in Figure 2. On *A. thaliana* (Naish et al. 2021), EasyCen (8 min) was slightly slower than QuarTeT (4 min), but included boundary refinement and architecture profiling absent from other tools. All methods produced highly concordant coordinates Table 2.

**TABLE 2:** Comparative evaluation of centromere localisation, computational efficiency, and repeat‐organisation profiling among EasyCen and existing frameworks in the mouse ( *Mus musculus* ) genome.

Chromosomes	CR (EasyCen)		CR (CentIER)		CR (RepeatOBserver)		CR (QuarTeT)	
1	1	11,200,000	1,666,666	7,333,333	1,250,000	10,755,000	1	18,297,098
2	1	12,300,000	2,500,000	10,999,999	1,250,000	12,230,000	1	12,750,115
3	1	5,400,000	4,500,000	5,999,999	1,250,000	5,790,000	1	7,515,674
4	1	2,900,000	1,500,000	2,999,999	1,250,000	3,410,000	1	2,863,421
5	1	9,000,000	1,833,333	7,666,666	1,250,000	9,060,000	1	8,959,464
6	1	6,000,000	500,000	4,499,999	1,250,000	5,155,000	1	5,056,111
7	1	18,100,000	7,166,666	14,833,333	1,250,000	17,570,000	1	21,837,313
8	1	13,000,000	2,333,333	10,666,666	1,250,000	12,445,000	1	16,402,358
9	1	11,500,000	4,666,666	9,833,333	1,250,000	10,850,000	1	11,312,881
10	1	8,500,000	7,500,000	8,999,999	1,250,000	8,545,000	1	8,426,160
11	1	11,000,000	1,833,333	8,166,666	1,250,000	10,835,000	1	14,989,817
12	1	4,700,000	1,500,000	3,499,999	1,250,000	3,390,000	1	531,114
13	1	11,100,000	0	1,499,999	1,250,000	10,755,000	1	11,218,671
14	1	11,200,000	1,833,333	9,666,666	1,250,000	11,060,000	1	11,462,625
15	1	8,700,000	1,666,666	6,833,333	1,250,000	7,775,000	1	10,755,289
16	1	14,200,000	2,000,000	10,499,999	1,250,000	12,110,000	1	16,718,885
17	1	12,300,000	2,333,333	10,666,666	1,250,000	11,795,000	1	13,045,040
18	1	10,200,000	1,666,666	7,333,333	1,250,000	9,430,000	1	11,282,742
19	1	8,000,000	1,666,666	6,833,333	1,250,000	7,140,000	1	13,455,614
X	1	3,500,000	1,500,000	3,499,999	1,250,000	3,720,000	170,392,577	170,815,025
Running time(s)	2,264		About 9 weeks		23,527		80,844	
Running time(m)	51		About 9 weeks		392		1,347	
Repeat‐organisation profiling	Yes		No		No		No	

On *M. musculus* (mhaESC assembly) (Chaudhry et al. 2025), EasyCen completed centromere identification and architecture profiling in 51 min, while QuarTeT required ~22.5 h, and CentIER nearly seven days. QuarTeT produced X‐chromosome coordinates that differed from established annotations, indicating its limitations in highly repetitive mammalian assemblies. RepeatOBserver showed a consistent offset (~1.25 Mb) likely due to sensitivity to telomeric repeats. EasyCen predictions showed strong concordance with annotations (coordinate concordance defined as overlap relative to the union span).

Across five additional diverse genomes (Figures [Link], [Link])—maize (Chen et al. 2023), rice (Shang et al. 2023), large yellow croaker (Cui et al. 2025), sandalwood (Peng et al. 2024), and green alga (Wang et al. 2024)—EasyCen predictions overlapped substantially with published annotations (Peng et al. 2024; Wang et al. 2024) or cytological landmarks (Cui et al. 2025; Chen et al. 2023; Shang et al. 2023). All analyses were completed in hours on a standard workstation. These findings indicate that EasyCen provides a computationally lightweight alternative for centromere localisation while additionally supporting exploratory repeat‐architecture analysis across diverse genomes.

### 2.3 Accuracy and Robustness of Repeat‐Pair‐Based Architecture Profiling

**FIGURE 3:**
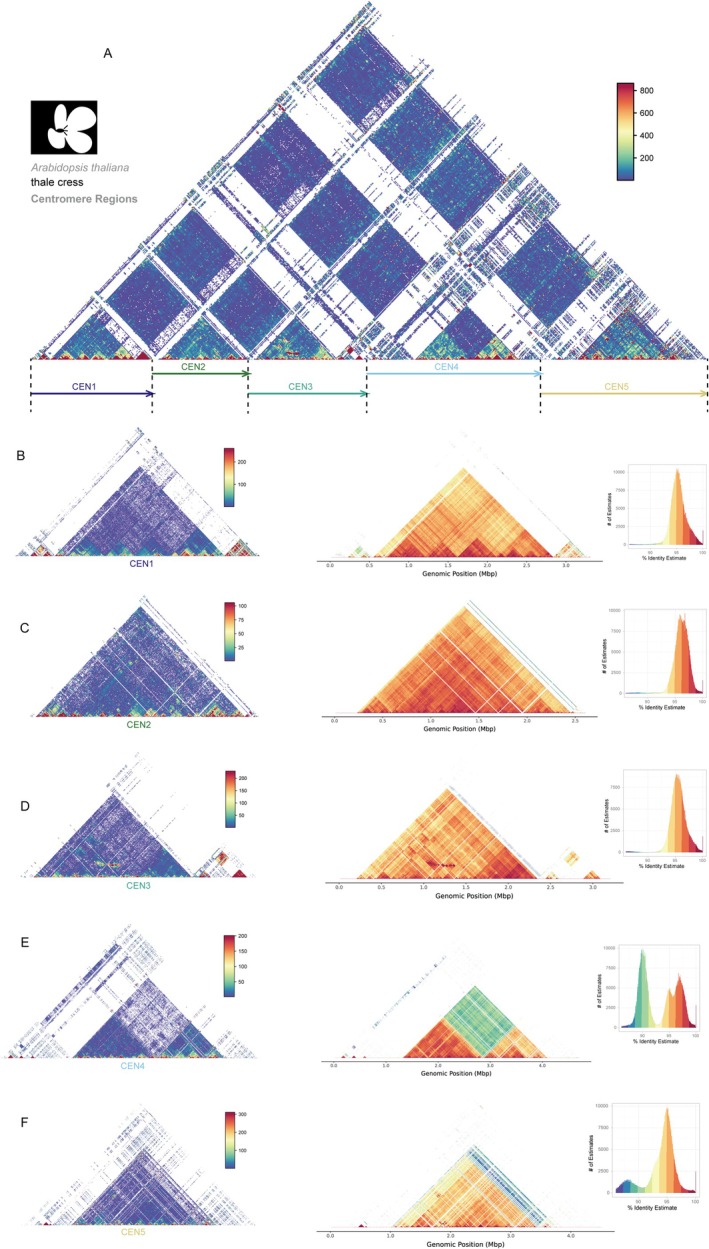
Centromere structure in *Arabidopsis thaliana* and comparison of EasyCen and MotDotPlot predictions. (A) Genome‐wide repeat k‐mer co‐occurrence map for the five centromeric regions (CEN1–CEN5). Contact frequencies are indicated using a colour scale. (B–F) Zoomed‐in views of individual centromeres. Left panels: Centromere structure and the structure predicted by EasyCen. Middle panels: Corresponding dot plots generated by MotDotPlot. Right panels: Histograms of sequence identity within each centromere, with colours consistent with those in MotDotPlot. Across all chromosomes, EasyCen predictions closely match MotDotPlot results, demonstrating the reliability of EasyCen in delineating centromeric structure.

The positional co‐occurrence matrix was reconstructed to delineate the centromeric architecture from combinatorial k‐mer interactions by EasyCen. On *A. thaliana* centromeres, the repeat‐pair framework showed strong agreement with similarity‐based methods in both boundary position and large‐scale structure (Figure 3). Notably, the repeat‐pair matrices detected local variations in repeat composition, homogenisation, and domain boundaries that were less apparent in conventional analyses—an advantage of using positional co‐occurrence rather than nucleotide alignment. The framework avoids computationally expensive repeat alignment and HOR profiling, making it scalable to large genomes. By focusing on architecture rather than identity, it enables cross‐species comparisons despite sequence divergence.

### 2.4 A Distinct Repeat‐Organisation Pattern in Mouse and Human Centromeres

**FIGURE 4:**
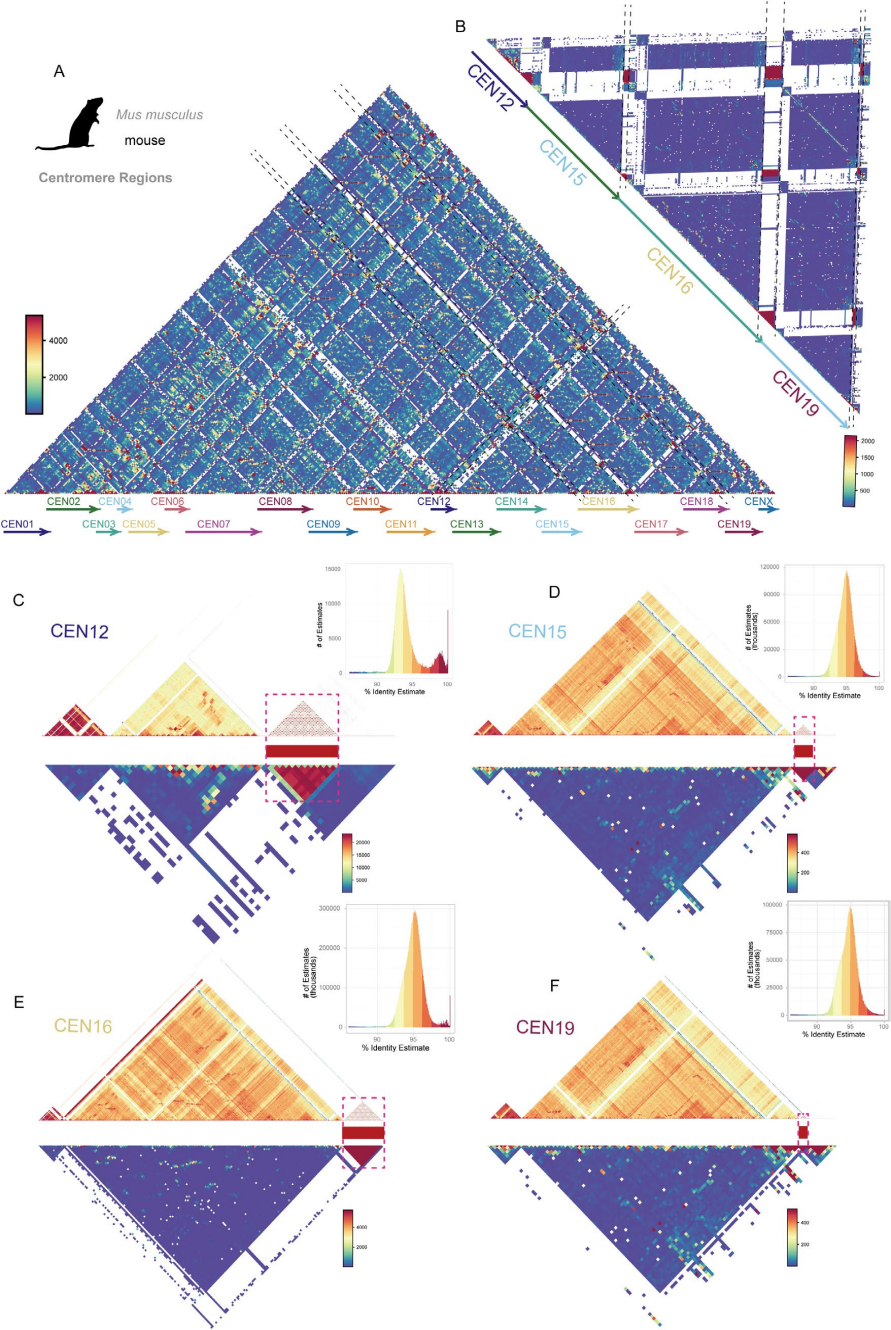
Centromere identification in mouse and the centromere‐specific regions detected by EasyCen. (A) Overview of mouse centromeric regions, highlighting several regions on specific chromosomes where repeat‐pair counts are markedly higher than in surrounding areas. (B) Magnified view of these centromere‐specific regions, including CEN12, CEN15, CEN16, and CEN19. (C–F) Bead‐like structures of the four centromeric regions. The upper panels show dot plots generated by MotDotPlot at 2 kb resolution, and the lower panels show the corresponding regions identified by EasyCen.

In the mouse genome, several chromosomes (12, 15, 16, 19) contained segments with unusually high combinatorial counts of repeat k‐mers (Figure 4). These regions displayed an alternating pattern of repeat‐enriched and non‐repetitive intervals, creating a ‘beads‐on‐a‐string’‐like configuration, which is most pronounced on chromosome 12.

**FIGURE 5:**
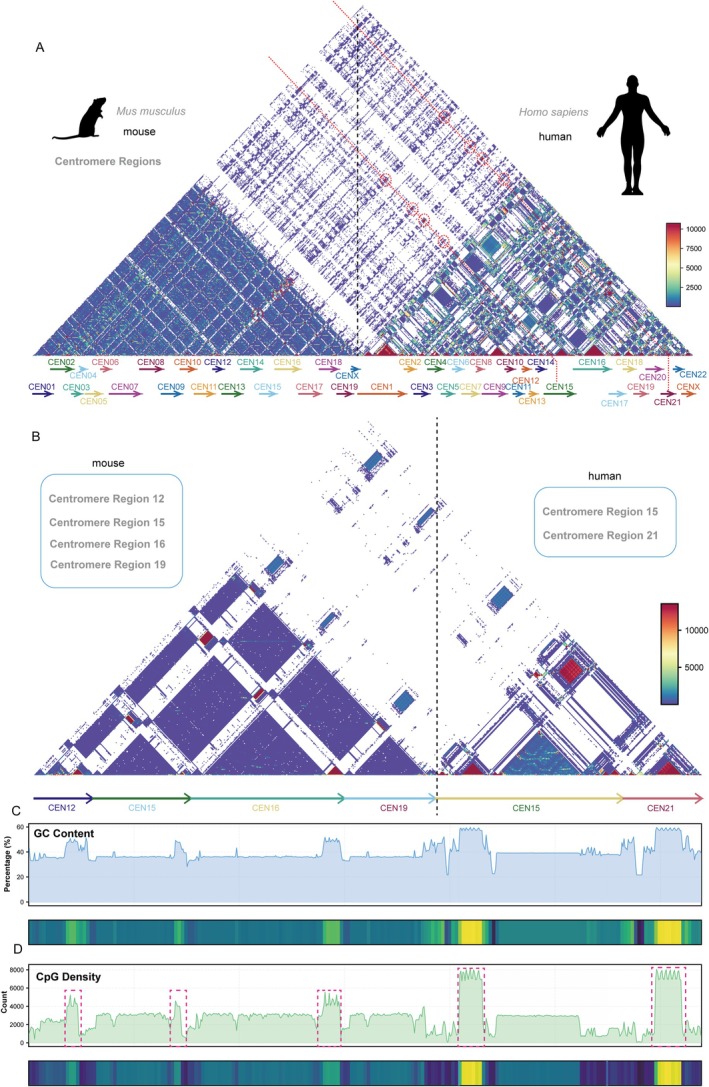
Comparative analysis of centromeric regions between mouse (on the left) and human (on the right). (A) Genome‐wide overview comparing mouse and human centromeres. (B) Centromere‐specific regions identified in mouse that correspond to centromeric regions on human chromosomes 15 and 21. (C) Comparison of GC content across these regions using 100‐kb windows, showing histograms in the upper panels and heatmaps in the lower panels. (D) Comparison of CpG density across the same regions using 100‐kb windows, showing histograms in the upper panels and heatmaps in the lower panels.

Human chromosomes 15 and 21 also showed this pattern (Figure 5), while other human centromeres did not. This observation suggests that related organisational patterns may occur across multiple mammalian lineages. The ‘beads‐on‐a‐string’‐like regions were enriched in GC content and CpG islands, indicating that sequence composition may contribute to structural heterogeneity within these centromeres. These compositional subdomains warrant further investigation.

### 2.5 Repeat Organisation and Centromere Identity

Our findings support the notion that centromere identity can be maintained by repeat organisation despite rapid sequence turnover. The periodicity, breadth, and clustering metrics capture emergent features of repeat amplification and homogenisation that persist across species. Recurrent tandem regularity, chromosomal confinement, and pan‐centromeric enrichment may reflect chromatin‐level constraints associated with centromere maintenance. The results also suggest that strict conservation of HOR sequence identity is not always required; positional architecture may itself provide structural cues. This framework underscores that centromere discovery can be driven by organisational features rather than by conserved motifs, providing a strategy for studying other rapidly evolving repetitive regions.

## 3 Methods and Materials

### 3.1 Data and Reproducibility

EasyCen is available at the GitHub repository (https://github.com/lvyunyunSCI/EasyCen.git). All scripts used to generate the analyses and figures presented in this study are also publicly available in the repository, enabling direct reproduction of centromere identification and repeat‐architecture analyses.

The reference genome assemblies used in this study, including thale cress ( *A. thaliana* ), mouse ( *M. musculus* ), human ( *H. sapiens* ), maise ( *Z. mays* ), rice ( *O. sativa* ), large yellow croaker (*L.s crocea*), sandalwood ( *S. album* ), and green alga ( *C. sorokiniana* ), were obtained from publicly available resources and original publications (Nurk et al. 2022; Chaudhry et al. 2025; Xie et al. 2025; Cui et al. 2025; Altemose et al. 2022; Chen et al. 2023; Shang et al. 2023; Wang et al. 2024).

Comprehensive documentation, executable workflows, parameter settings, and step‐by‐step tutorials are provided through the EasyCen project page to facilitate full reproducibility. All analyses presented in this study can be reproduced directly using the supplied pipeline and example commands.

### 3.2 Methodological Overview

EasyCen uses rolling k‐mer hashing to compute canonical k‐mers. Default parameters were as follows: k = 21, window size 100 kb, step 10 kb, periodicity ≥ 0.5, breadth≥ 50%, clustering ≥ 0.5. Filtering removes low‐complexity and over‐abundant k‐mers based on count, entropy, spacing, and optional curated repeat libraries. Outputs include bedGraph tracks for k‐mer density, GC content, CpG density, and unique k‐mer proportion. Gaussian boundary refinement delineates centromeres; chromosome‐level parallelism and combinatorial k‐mer pair profiling enable the quantitative characterisation of internal repeat organisation.

### 3.3 Architecture Comparison

The EasyCen kmer‐pairs command generated repeat‐pair combinatorial profiles. Orthogonal validation used moddotplot version 0.9.9 (2 kb window) (Sweeten et al. 2024). Concordance was assessed by comparing module segmentation, dominant repeat enrichment zones, and local complexity trends.

### 3.4 Cross‐Species Analysis

Positional co‐occurrence matrices were computed for all mouse chromosomes. High‐frequency regions were confirmed with moddotplot. The human T2T genome was included; the Y chromosome was excluded. GC and CpG island densities were calculated in 100‐kb sliding windows and compared to chromosome‐wide backgrounds.

## 4 Conclusions

We present EasyCen, a lightweight annotation‐independent framework for centromere identification and repeat‐organisation profiling in T2T genomes. By combining k‐mer‐based features with repeat‐pair profiling, the method achieves accurate boundary detection and reveals internal organisation without relying on prior annotations. Benchmarking across plant and mammalian genomes demonstrated high accuracy and improved computational efficiency, particularly for large genomes. The application of EasyCen uncovered a ‘beads‐on‐a‐string’‐like repeat pattern in mouse and human centromeres, suggesting the existence of links between sequence composition and centromeric structural heterogeneity. As T2T assemblies proliferate, EasyCen provides a practical tool for comparative and evolutionary analysis of centromeres. More broadly, this work offers a framework to investigate how repeat organisation drives centromere evolution and shapes chromosome architecture across eukaryotes.

## Author Contributions

Yunyun Lv and Xidong Mu conceived and supervised the study. Yunyun Lv, Yanping Li, and Jia Li performed the experiments and data analyses. Yunyun Lv and Jia Li drafted the manuscript. All authors contributed to revising the manuscript and approved the final version.

## Funding

This work was supported by the National Freshwater Genetic Resource Center, FGRC18537. Guangdong Provincial Special Fund for Modern Agriculture Industry Technology Innovation Teams, 2024CXTD26. Sichuan Provincial Funding for Freshwater Fish Innovation, SCCXTD‐2026‐15. Neijiang Normal University Special Project, X26B0011. Open Fund Projects for the Fishes. Conservation and Utilization in the Upper Reaches of the Yangtze River Key Laboratory of Sichuan Province, NJTCSC23‐1. Open Fund Projects for the Key Laboratory of Freshwater Fish Reproduction and Development, FFRD‐2022‐03.

## Conflicts of Interest

The authors declare no conflicts of interest.

## Supporting information


**Figure S1:** Genome‐wide centromere identification in the maize ( *Zea mays* ) T2T genome using EasyCen.

**Figure S2:** Genome‐wide centromere identification in the rice ( *Oryza sativa* ) T2T genome using EasyCen.

**Figure S3:** Genome‐wide centromere identification in the large yellow croaker ( *Larimichthys crocea* ) T2T genome using EasyCen.

**Figure S4:** Genome‐wide centromere identification in the sandalwood ( *Santalum album* ) T2T genome using EasyCen.

**Figure S5:** Genome‐wide centromere identification in the near T2T genome of green alga ( *Chlorella sorokiniana* ) using EasyCen.

## Data Availability

The data that support the findings of this study are openly available in https://github.com at https://github.com/lvyunyunSCI/EasyCen.
