# Supplementary figures and images for: EasyCen: A Lightweight Framework for Centromere Localisation and Repeat‐Organisation Profiling in Telomere‐to‐Telomere Genomes
Source: Mol Ecol Resour. 2026 Jul 2;26(5):e70176. doi: 10.1111/1755-0998.70176 (PMC13324764; doi:10.1111/1755-0998.70176)

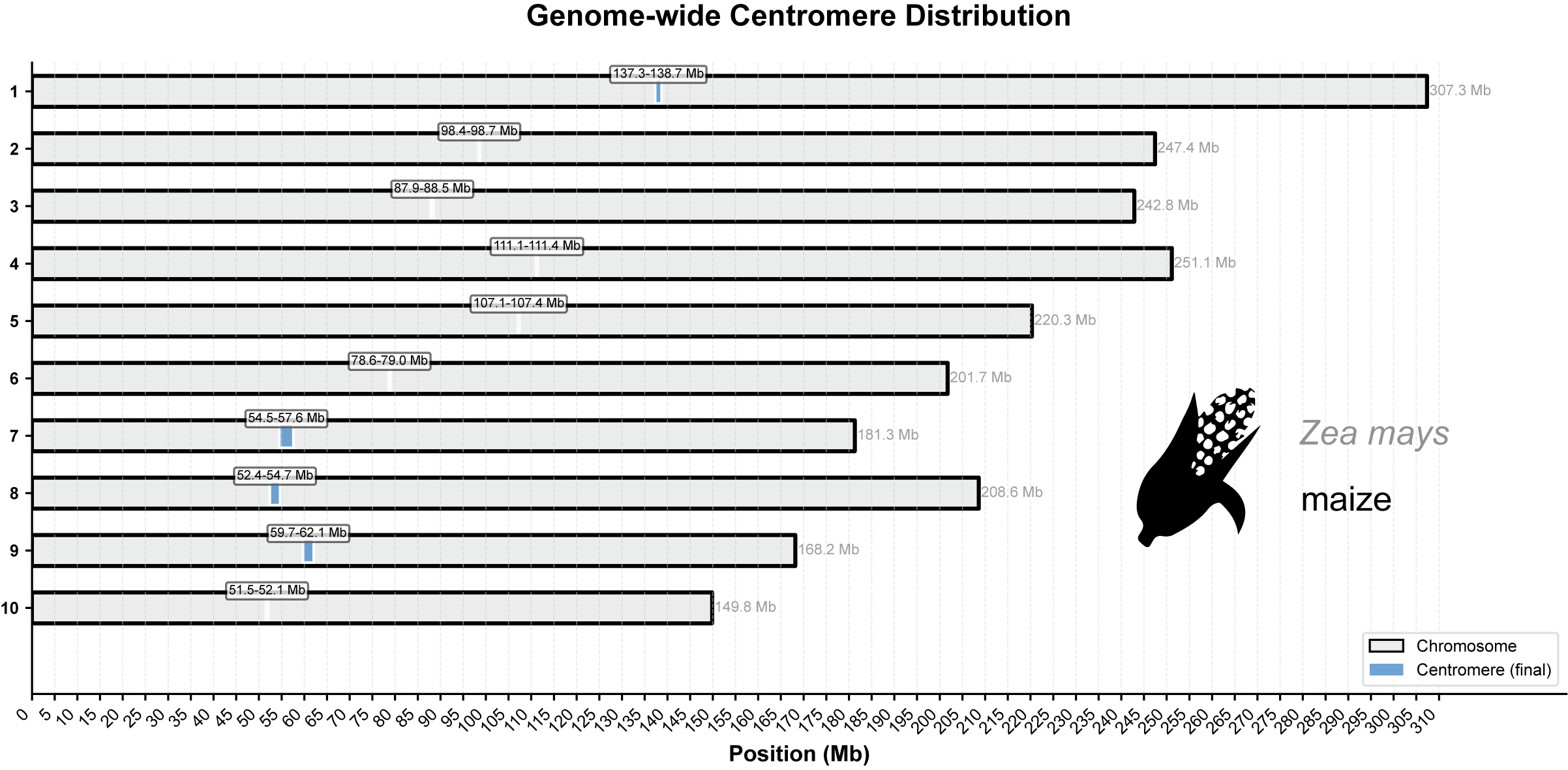

Supplement: Supplementary file 1 — Figure S1: Genome‐wide centromere identification in the maize ( Zea mays ) T2T genome using EasyCen. [file MEN-26-e70176-s002.png]

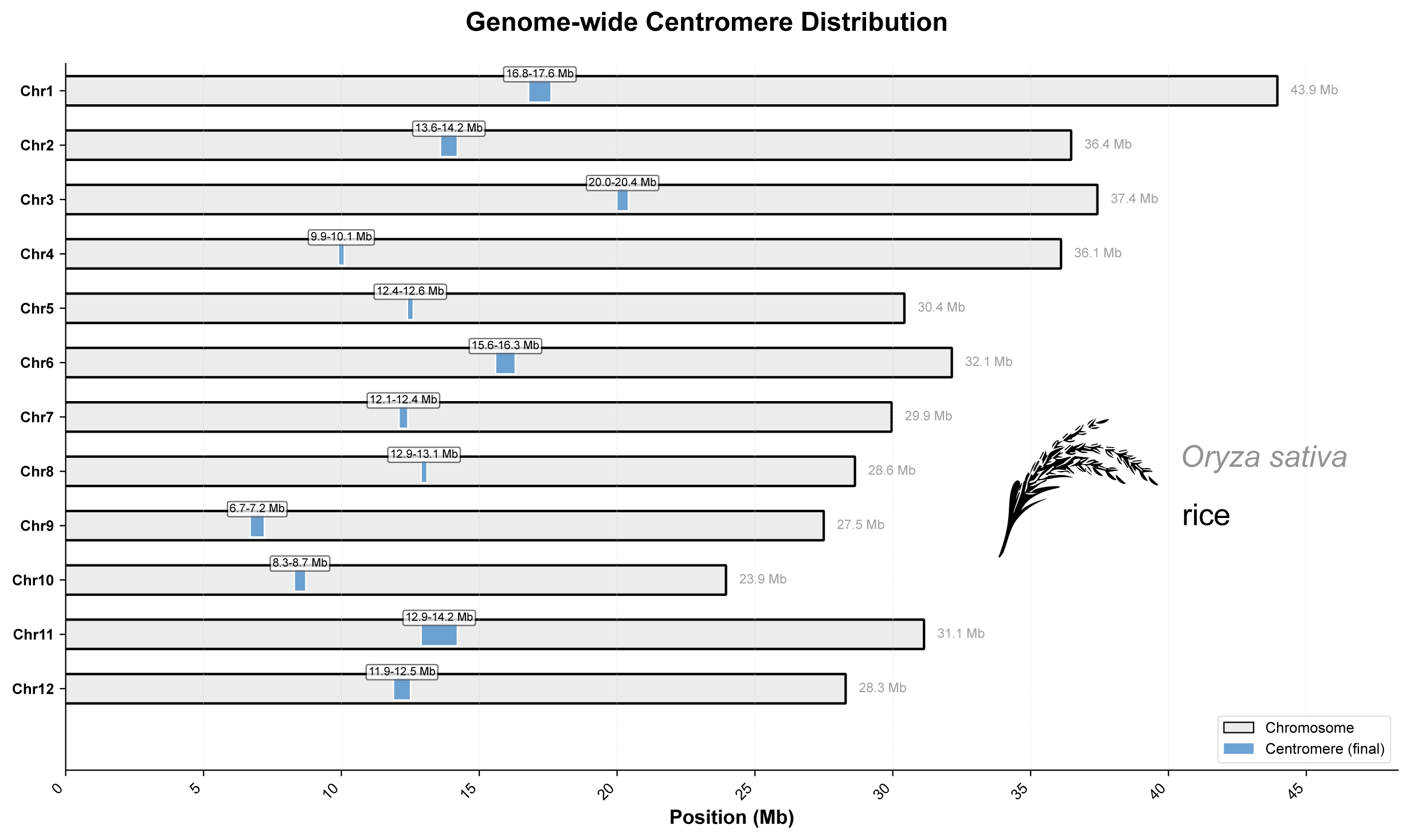

Supplement: Supplementary file 2 — Figure S2: Genome‐wide centromere identification in the rice ( Oryza sativa ) T2T genome using EasyCen. [file MEN-26-e70176-s001.png]

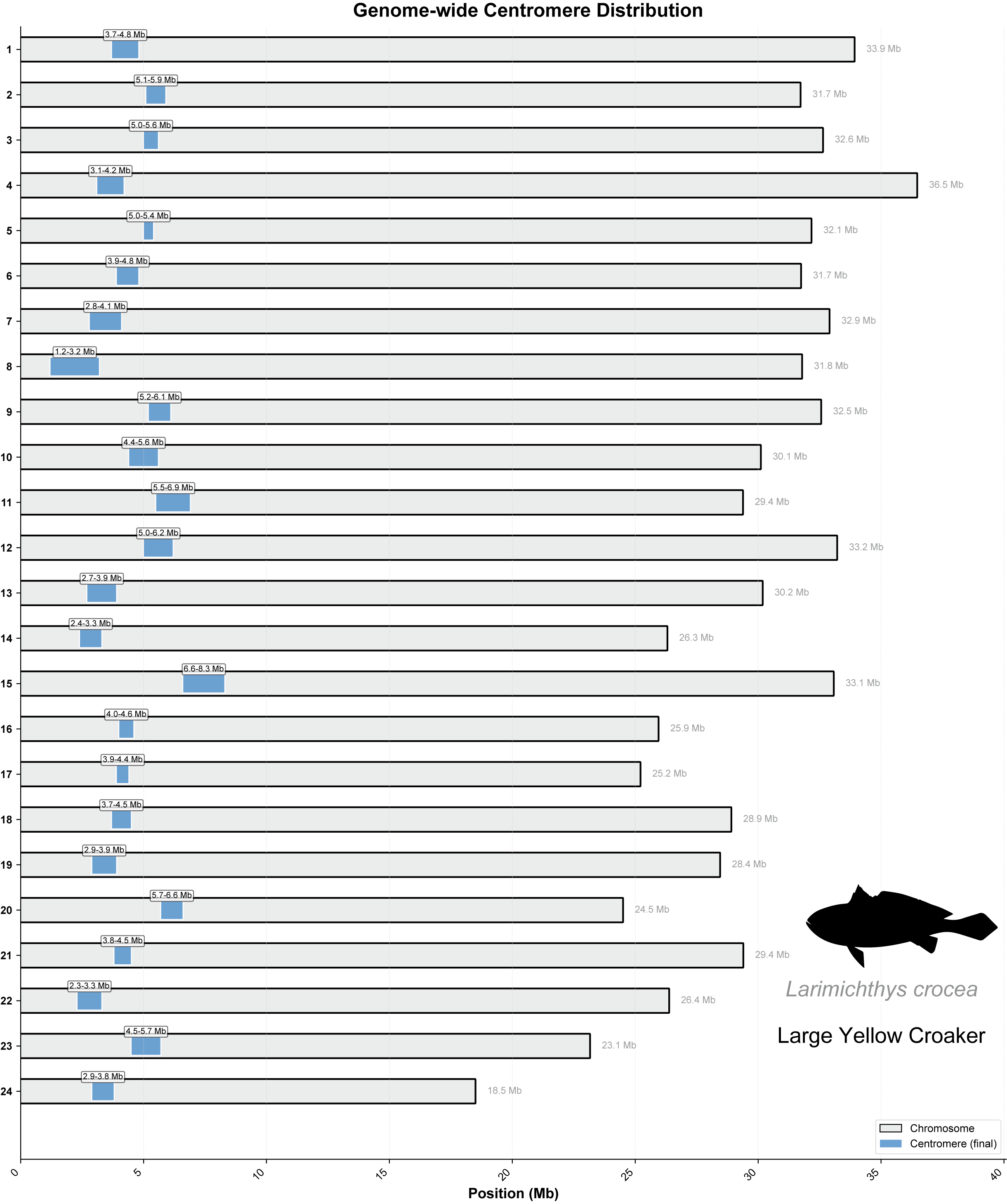

Supplement: Supplementary file 3 — Figure S3: Genome‐wide centromere identification in the large yellow croaker ( Larimichthys crocea ) T2T genome using EasyCen. [file MEN-26-e70176-s004.png]

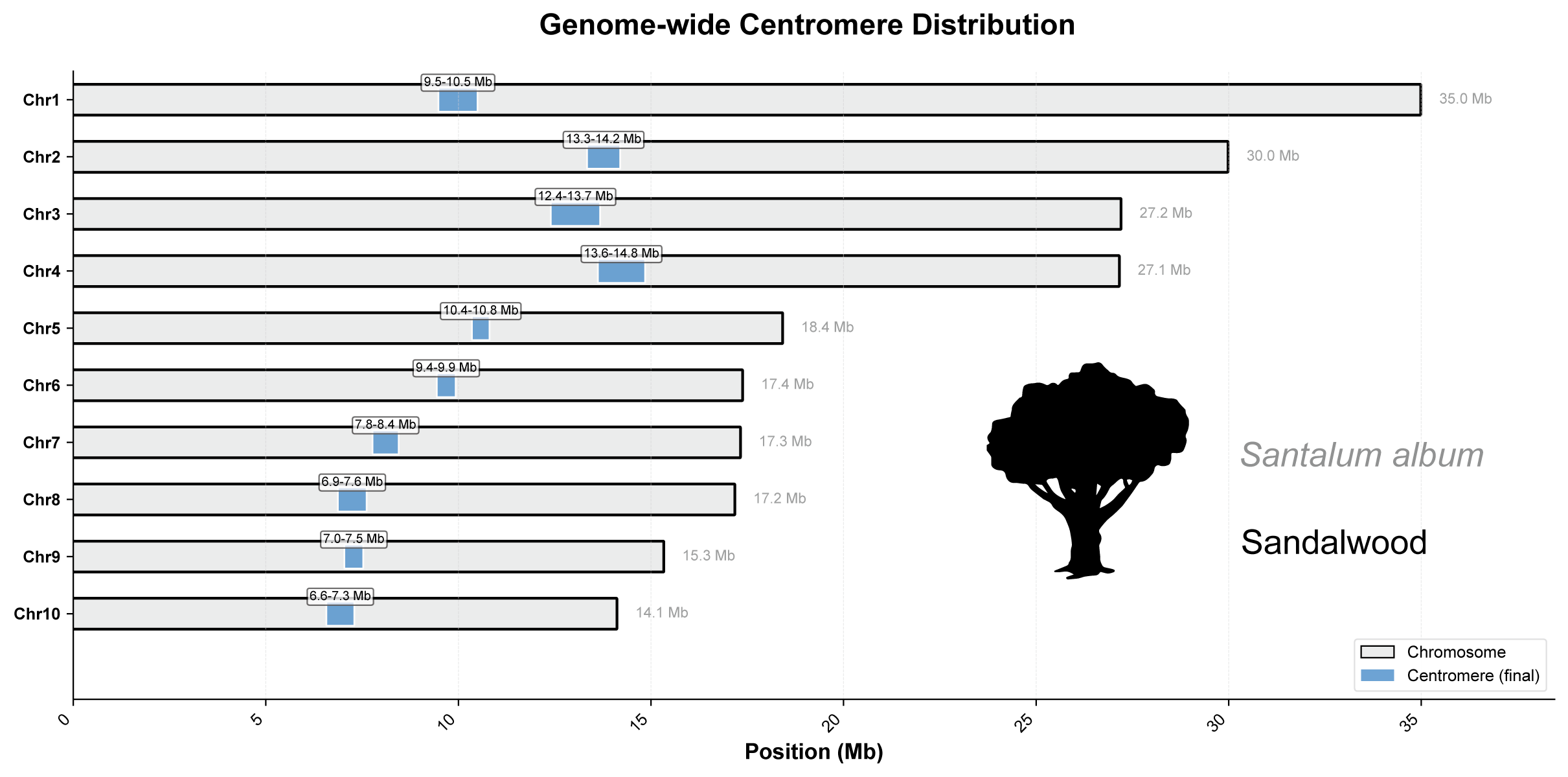

Supplement: Supplementary file 4 — Figure S4: Genome‐wide centromere identification in the sandalwood ( Santalum album ) T2T genome using EasyCen. [file MEN-26-e70176-s005.png]

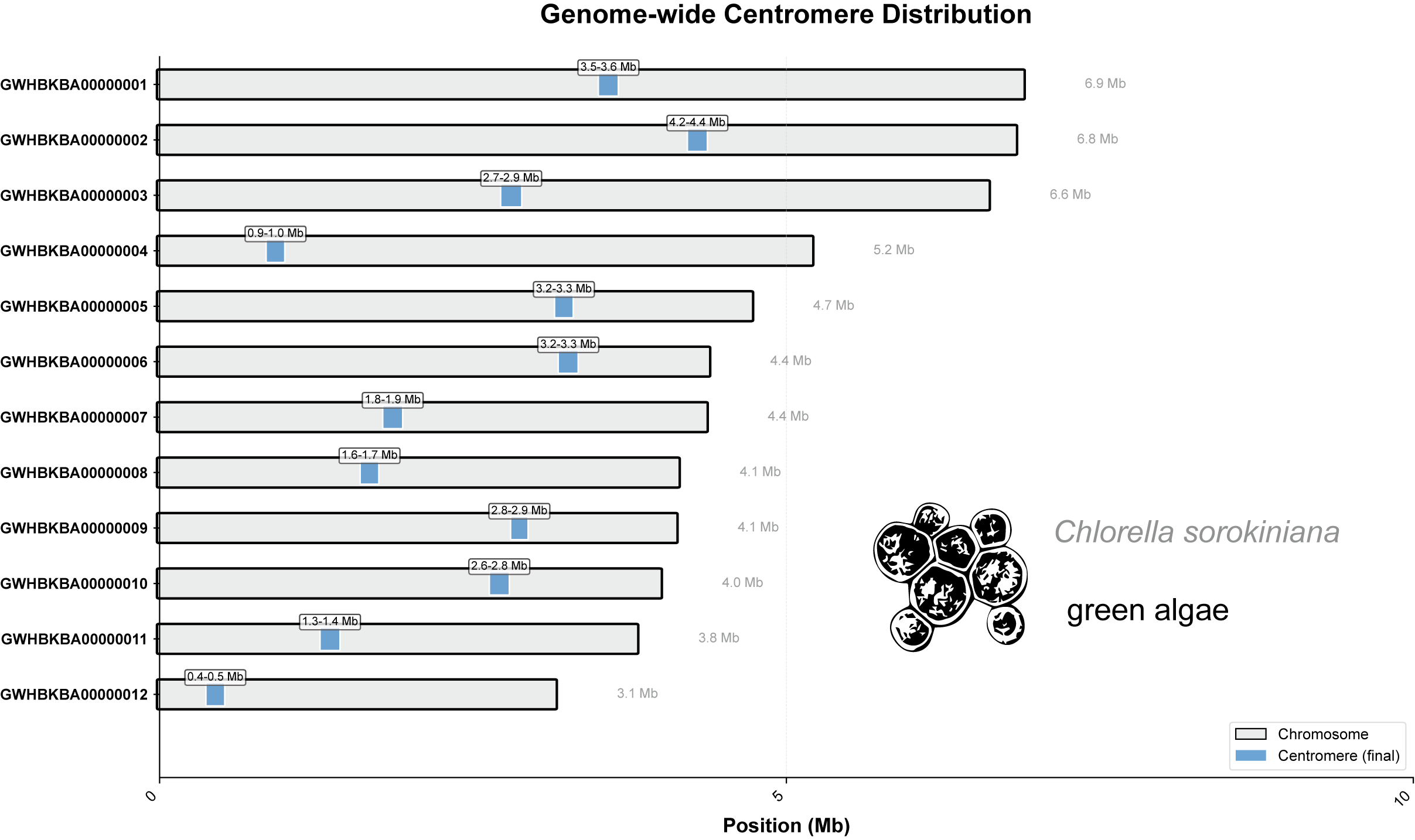

Supplement: Supplementary file 5 — Figure S5: Genome‐wide centromere identification in the near T2T genome of green alga ( Chlorella sorokiniana ) using EasyCen. [file MEN-26-e70176-s003.png]
